# The SGLT family—sodium‐glucose transporters with roles beyond glucose and the kidney

**DOI:** 10.1111/jcmm.18152

**Published:** 2024-03-06

**Authors:** David Mathew, Sean Davidson, Derek Yellon

**Affiliations:** ^1^ The Hatter Cardiovascular Institute University College London London UK

The inhibition of urinary sodium‐glucose reuptake via the SGLT2 transporter in the proximal tubule of the nephron was initially intended as a treatment for diabetes. However, the unexpected efficacy of SGLT2‐inhibitors in improving both cardiovascular and renal outcomes has positioned this class of pharmacological agents centre‐stage in the burgeoning era of preventative medicine.^1^ There are six named members of the Sodium‐Glucose co‐Transporter (SGLT) family, some of which have been detected in tissues other than the kidney and are responsible for the transport of monosaccharides other than glucose.^2^ The diversity of both transporters and substrates raises the question of what the roles of these SGLTs are, and whether they are interesting clinical targets. The SGLT2‐inhibitors or ‘gliflozins’ are not fully selective for the SGLT2 isoform and animal models have demonstrated that Empagliflozin, for example, mediates improvement of hyperglycaemic endothelial dysfunction via both SGLT1 and 2.^3^, ^4^ Similarly, in vivo studies indicate that Canagliflozin may exert some cardioprotective effects via SGLT1.^5^ A study of the function of the remaining SGLTs may reveal some of the mechanisms underlying the efficacy of SGLT2 inhibitors. Furthermore, investigating the selective inhibition of all six SGLTs and their associated monosaccharides may identify novel treatments, enabling identification of patient groups with capacity to benefit from targeted pharmacotherapy.

The primary role of SGLT1, is glucose, sodium and water reabsorption at the small intestine, however it has also been detected in disparate cell populations including: myocardium, endometrium and lymphocytes.^2^ SGLT1 expression is elevated in human myocardium in the context of ischaemic and diabetic cardiomyopathy with selective inhibition improving cardiac apoptosis and fibrosis in diabetic animal models.^6^, ^7^ The expression of SGLT1 can also be detected in lymphocyte populations, indicating a potential role in immune modulation which is supported by evidence that SGLT1 deficiency impairs response to bacterial infection.^8^ However, development of a selective inhibitor of this transporter has not overcome the challenge of diarrhoea and dehydration induced by intestinal SGLT1 inhibition. Clinical evaluation of Sotagliflozin, a dual SGLT1/2 inhibitor has been limited by heterogeneity of Phase 3 data and has not yet extended to patients without Type 2 Diabetes.^9^ As such, insight into any additional benefit of combined SGLT1 and SGLT2 inhibition is currently lacking. Furthermore, volume depletion and reduction in estimated Glomerular Filtration Rate (eGFR) remain a concern.^10^

Targeting of SGLTs 3,4 and 5 may further the understanding and treatment of metabolic disease including diabetes and obesity. Although not a transporter per se, but rather a glucose sensor expressed in cholinergic neurons of the enteric nervous system, SGLT3 is implicated in mediation of gastric emptying. Furthermore it is downregulated in obesity and its activity stimulates Glucagon Like Peptide 1 (GLP‐1) secretion.^11^, ^12^ Whole exome sequencing has identified SGLT4, which is primarily a mannose transporter, as a target of interest in the pathogenesis of diabetic retinopathy.^2^ SGLT4 expression is also increased in kidney, pancreatic and colorectal tumours, perhaps hinting at a role in oncogenesis.^2^

SGLT5 is the primary transporter responsible for fructose reuptake in the proximal tubule, analogous to the role of SGLT2 in glucose reuptake. High dietary fructose intake in the form of high fructose corn syrup is linked with the metabolic syndrome, chronic kidney disease and cardiovascular morbidity.^13^ Its harmfulness is augmented by a lack of negative feedback regulation in the ‘fructolytic’ pathway giving rise to intracellular hypertriglyceridemia, uric acid generation and an associated inflammatory milieu.^13^ Inhibition of renal fructose re‐uptake may therefore present an appealing therapeutic strategy.

The reuptake of 1,5‐AG (1,5‐Anhydroglucitol), a monosaccharide present in almost all foods is also mediated via SGLT5 at the proximal tubule. Knowledge regarding the physiological role of 1,5‐AG is limited, however accumulation within neutrophils causes dysfunction and depletion in two rare congenital neutropenia's: Glycogen Storage Disease 1b (GSD1b) and Severe Congenital Neutropenia Type 4.^14^ Treatment of GSD1b in humans with Empagliflozin has been successful as SGLT2 inhibition indirectly promotes urinary loss of 1,5‐AG at the expense of glucose reuptake.^14^

SGLT6, which is expressed in the kidney, small intestine and brain, is responsible for transport of the monosaccharide myo‐inositol, which is of interest in neurological disease.^15^ Myo‐inositol transport enabled by SGLT6 may protect against development of cerebral oedema in ischaemic stroke, and the gene has been implicated in the ‘infantile convulsions and paroxysmal dyskinesia syndrome’.^2^, ^16^ SGLT6 is also thought to be active in immune‐modulation in the context of rheumatological disease such as Systemic Lupus Erythematosus.^17^

In conclusion, the use of SGLT2 inhibitors in clinical practice has provided improvement in cardiovascular and renal outcomes of an unexpected magnitude, but understanding of the underlying physiological mechanisms is incomplete. The role of the other five members of the SGLT family and potential utility of selective inhibition is not known and has not been extensively explored. The existing literature hints at benefits in the fields of diabetes, obesity, nephrology, cardiology, immunity and neurology and should be investigated in more detail.

## AUTHOR CONTRIBUTIONS

**David Mathew:** Data curation (equal); formal analysis (equal); investigation (equal); writing – original draft (equal); writing – review and editing (equal). **Sean Davidson:** Project administration (equal); writing – review and editing (equal). **Derek Yellon:** Conceptualization (equal); project administration (equal); writing – review and editing (equal).

## FUNDING INFORMATION

Funded by British Heart Foundation Grant number PG/21/10798.

## CONFLICT OF INTEREST STATEMENT

The authors confirm that there are no conflict of interest.

## Data Availability

Data sharing not applicable—no new data generated, or the article describes entirely theoretical research.
